# Spatiotemporal Variations in Lung Cancer Mortality in China between 2006 and 2012: A Multilevel Analysis

**DOI:** 10.3390/ijerph13121252

**Published:** 2016-12-16

**Authors:** Yunning Liu, Thomas Astell-Burt, Jiangmei Liu, Peng Yin, Xiaoqi Feng, Jinling You, Andrew Page, Maigeng Zhou, Lijun Wang

**Affiliations:** 1 National Center for Chronic and Noncommunicable Disease Control and Prevention, Chinese Center for Disease Control and Prevention, Beijing 100050, China; liuyunning0723@163.com (Y.L.); jiangmei.liu@foxmail.com (J.L.); yinpengcdc@163.com (P.Y.); youjinling888@163.com (J.Y.); maigengzhou@126.com (M.Z.); 2 Population Wellbeing and Environment Research Lab (PowerLab), Faculty of Social Sciences, University of Wollongong, Wollongong, NSW 2522, Australia; thomasab@uow.edu.au (T.A.-B.); xfeng@uow.edu.au (X.F.); 3 School of Health and Society, Faculty of Social Sciences, University of Wollongong, Wollongong, NSW 2522, Australia; 4 Early Start Research Institute, Faculty of Social Sciences, University of Wollongong, Wollongong, NSW 2522, Australia; 5 School of Medicine, University of Western Sydney, Sydney, NSW 2006, Australia; A.Page@westernsydney.edu.au

**Keywords:** lung cancer, mortality, geographical variation, temporal trends, urbanization level

## Abstract

We investigated temporal trends and geographical variations in lung cancer mortality in China from 2006 to 2012. Lung cancer mortality counts for people aged over 40 years were extracted from the China Mortality Surveillance System for 161 disease surveillance points. Negative binomial regression was used to investigate potential spatiotemporal variation and correlations with age, gender, urbanization, and region. Lung cancer mortality increased in China over the study period from 78.77 to 85.63 (1/100,000), with higher mortality rates evident in men compared to women. Median rate ratios (MRRs) indicated important geographical variation in lung cancer mortality between provinces (MRR = 1.622) and counties/districts (MRR = 1.447). On average, lung cancer mortality increased over time and was positively associated with county-level urbanization (relative risk (RR) = 1.15). Lung cancer mortality seemed to decrease in urban and increase in rural areas. Compared to the northwest, mortality was higher in the north (RR = 1.98), east (RR = 1.87), central (RR = 1.87), and northeast (RR = 2.44). Regional differences and county-level urbanization accounted for 49.4% and 8.7% of provincial and county variation, respectively. Reductions in lung cancer mortality in urban areas may reflect improvements in access to preventive healthcare and treatment services. Rising mortality in rural areas may reflect a clustering of risk factors associated with rapid urbanization.

## 1. Introduction

Lung cancer is the leading cause of cancer-related mortality worldwide. It accounts for 19.4% (1.6 million) of total cancer cases in 2012 [1]. In China, lung cancer has risen to be the fourth leading cause of death [2]. The Global Burden of Disease Study 2013 [2] has shown an increasing trend in lung cancer mortality from 36.04 per 100,000 in 1990 to approximately 40.41 per 100,000 two decades later. Despite investments in initiatives and interventions on tobacco smoking behavior, lung cancer remains a major public health problem in China [3]. Tobacco smoke is likely to be the major cause of lung cancer mortality, but other environmental factors may also play a role, such as air pollution, with some research suggesting ambient concentrations of Particulate Matter 2.5 are associated with small but measurable increases in lung cancer mortality [4,5].

The prevalence of smoking and air pollution, as major determinants of lung cancer, varies substantially between rural and urban areas in China. It is therefore likely that lung cancer incidence and mortality also vary by urbanization level [6,7]. However, few studies have investigated geographical variations in lung cancer mortality and correlations with urbanization in China. If there are differences in lung cancer mortality in different regions, it is likely that the epidemiology of risk factors and the provision and access to preventive healthcare and treatment service allocations will also differ spatially. Revealing geographic differences in lung cancer mortality is important from a policy perspective and, through spatial targeting, can enhance the efficiency of promotion and prevention messages and service planning.

Accordingly, the aim of this study was to investigate temporal trends and geographical variations in lung cancer mortality in Mainland China from 2006 to 2012. The study used unique data from a nationally representative sample of mortality spanning all 31 provinces: the China Mortality Surveillance System. The key objectives of this study were as follows:

(i) to investigate temporal trajectories in lung cancer mortality across China from 2006 to 2012;
(ii) to explore whether there are geographical differences in lung cancer mortality rates and to determine if those differences vary over time;
(iii) to assess the degree of correlation between lung cancer mortality and urbanization.

## 2. Materials and Methods

### 2.1. Design

A time-series analysis of a nationally representative mortality surveillance by geographic area was conducted in order to investigate changes and area differentials in lung cancer mortality rates over the 2006–2012 period.

### 2.2. Data

The International Classification of Diseases 10 (ICD-10) was used to define lung cancer mortality [8] in the China Mortality Surveillance System. The relevant codes included C33 (malignant neoplasm of trachea) and C34 (malignant neoplasm of bronchus and lung). Mortality counts were cross-classified by 5-year age group (>40 years), gender, year (2006 to 2012 inclusive) and Disease Surveillance Point (DSP). The DSP system comprises 161 counties or districts across all 31 provinces, municipalities, and autonomous regions in Mainland China, providing coverage of approximately 73 million people. A DSP is a county in a rural area or a district in an urban area. Counties or districts are the administrative units in China, and the geographic areas covered by each DSP were consistent over the study period. Previous work has demonstrated the representativeness of the DSP system [9,10].

County- or district-level (i.e., DSP) population counts cross-classified by 5-year age group and gender were obtained from the Chinese census in 2000 and 2010. These counts were used as a reference population for calculating age-standardized lung cancer mortality rates and as a population offset in regression models. To account for population change across the study period, census data from 2000 was used to calculate the annual rate of change in population for each of the 161 DSPs up to 2010. The total population for each DSP in the years 2006–2012 was then estimated assuming a uniform rate of change across the time period, in line with the methodology used in the Global Burden of Disease Study 2010 [11].

As the focus of the study was to describe geographical variation, two variables were selected for this analysis: (i) regional affiliation and (ii) urbanization level.

All DSPs were classified according to their regional affiliation by the China National Bureau of Statistics: east (Shanghai, Shandong, Jiangsu, Anhui, Jiangxi, Zhejiang and Fujian), north (Beijing, Tianjin, Hebei, Shanxi and Inner Mongolia), central (Hubei, Hunan and Henan), south (Guangdong, Guangxi and Hainan), southwest (Chongqing, Sichuan, Guizhou, Yunnan and Tibet), northwest (Shaanxi, Gansu, Ningxia, Xinjiang and Qinghai), and northeast (Heilongjiang, Jilin and Liaoning).

Urbanization level is often considered a proxy of socioeconomic circumstances and development [12]. In this study, urbanization was defined as the proportion of residents in each DSP classified as living within urban areas in the 2010 census. Tertiles of urbanization were used to examine for potentially curvilinear associations with lung cancer mortality.

### 2.3. Statistical Analysis

To investigate whether lung cancer mortality increased over time in China, age-standardized lung cancer mortality rates for each year across the study period (2006–2012) were calculated overall and separately by gender, urbanization, and region. Regional variation in age-standardized lung cancer mortality was also mapped using a Geographic Information System (ArcGIS Desktop 10, ESRI, Redlands, CA, USA).

Multilevel models (or hierarchical models) were selected to analyze the lung cancer mortality time-series to adjust parameters and standard errors for repeated measures of the same areas over time [13]. A three-level model was fitted, with the DSP/gender/5-year age group cross-classification at Level 1, county at Level 2, and province at Level 3. Negative binomial regression was selected to account for over-dispersion of the mortality counts. The natural logarithm of the equivalently classified denominator counts was fitted as an offset to account for local population distribution.

An initial model included fixed effects for age group, gender, and year. Potential curvilinear trajectories in lung cancer mortality through time were investigated using linear and polynomial terms. A random slope was fitted between the year variable and DSPs to examine whether trajectories were geographically consistent or specific to particular counties/districts. Further exploration of geographic variation was examined through the calculation of median rate ratios (MRRs). The MRR [14] is calculated using the same equation as the median odds ratio and has a similar interpretation, and is derived from the county/district and provincial variances. Values equal to 1 suggest no geographic variation in the outcome variable, whereas values above 1 indicate the necessity of taking context into account.

Dummy variables for the urbanization tertiles and regions were added as fixed effects. The percentage change in variance (PCV) was calculated for counties/districts and provinces after adding these variables. Interaction terms between regional variables and time were fitted to investigate whether regional inequalities and lung cancer mortality rates varied across the study period, and interaction terms between urbanization and time were also fitted to investigate whether urbanization differences and lung cancer mortality rates varied across the study period. All fixed effect parameters were exponentiated to relative risk (RR) and 95% confidence intervals (95% CI). All statistical analyses were conducted in Centre for Multilevel Modelling (MLwiN v2.30, University of Bristol, Bristol, UK) [15].

## 3. Results

### 3.1. Mortality and Proportion of Lung Cancer

**Figure 1:**
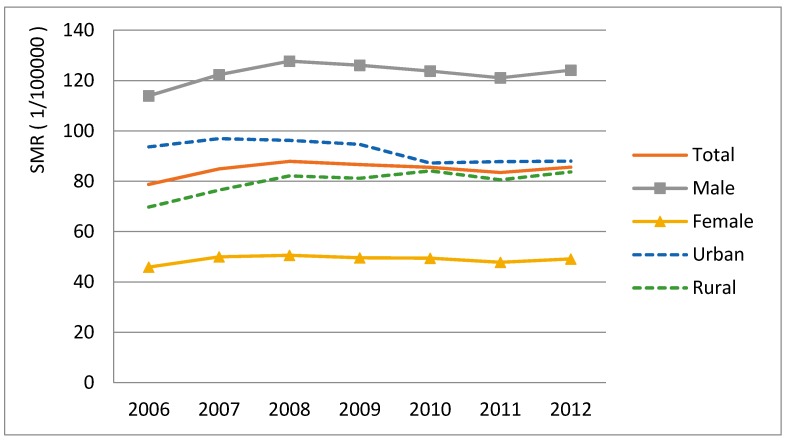
Lung cancer mortality trends during 2006–2012. SMR = Standardized mortality rate.

The overall age-standardized lung cancer mortality among people aged 40 years or older increased 8.71% (from 78.77 to 85.63 per 100,000) from 2006 to 2012 (Figure 1). Men had a consistently higher mortality rate than women throughout the years. The disparity between urban and rural was also notable. Lung cancer mortality during the study period decreased by 6% (from 93.67 to 88.02 per 100,000) in the urban areas, but increased by 20% (from 69.79 to 83.76 per 100,000) in rural areas. The gap between urban and rural narrowed over time.

**Figure 2:**
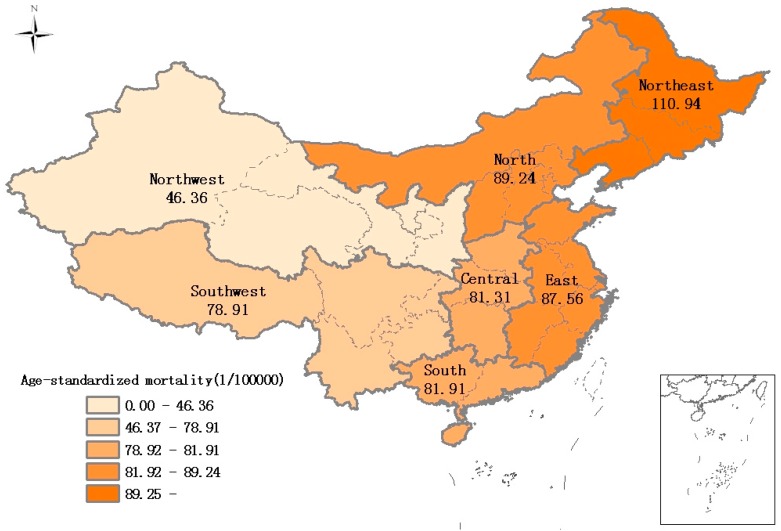
Distribution of age-standardized mortality of lung cancer in China.

The regional distribution of age-standardized lung cancer mortality (averaged from 2006 to 2012 inclusive) is shown in Figure 2. Mortality rates were highest in the northeast region and lowest in the northwest.

### 3.2. Multilevel Modeling of Lung Cancer Mortality

Table 1 illustrates the results of the multilevel modeling of the lung cancer mortality outcome. There was an overall increase in lung cancer mortality between 2006 and 2012 (RR = 1.052, 95% CI 1.034, 1.071), though the square term indicates that this increase was tailing off (RR = 0.992, 95% CI 0.990, 0.994). Lung cancer mortality was significantly higher among men (RR = 0.399, 95% CI 0.393, 0.406) and increased with age (RR = 1.505, 95% CI 1.502, 1.508). Lung cancer mortality varied between different provinces (MRR = 1.622) and different DSPs (MRR = 1.447).

Part of the province-level variation (3.50%) and DSP-level variation (10.00%) in lung cancer mortality was explained by the adjustment for urbanization level in Model 2. Compared with low urbanization, highly urbanized areas had higher lung cancer mortality (RR = 1.164, 95% CI 1.021, 1.328). There was no significant difference in mortality between low and moderately urbanized areas (RR = 1.045, 95% CI 0.911, 1.199). Lung cancer mortality varied significantly between different provinces (MRR: 1.608) and DSPs (MRR: 1.420).

Model 3 shows that much of the provincial variation was explained by regional differences (45.92%) that were illustrated in Figure 2. Compared to the northwest areas, the relative risk of lung cancer mortality was higher in people living in the north (RR = 1.982, 95% CI 1.212, 3.241), east (RR = 1.866, 95% CI 1.187, 2.935), central (RR = 1.866, 95% CI 1.072, 3.250) and northeast (RR = 2.442, 95% CI 1.400, 4.262) areas. There was no significant difference among northwest, south (RR = 1.361, 95% CI 0.771, 2.402), and southwest areas (RR = 1.026, 95% CI 0.626, 1.682). After full adjustment, lung cancer mortality still varied significantly between different provinces (MRR: 1.410) and DSPs (MRR: 1.423). Overall, 49.42% of the variation between provinces and 8.67% of the variation between DSPs in lung cancer mortality were explained by geographical region and urbanization level.

A small but statistically significant variance in DSP slopes through time indicated some spatiotemporal variation. Negative covariance (−0.012) between intercepts and slopes indicated a narrowing of DSP-level inequality in lung cancer mortality across the study period.

## 4. Discussion

### 4.1. Key Findings

The key findings from the study are that lung cancer mortality varies significantly across regional, provincial, and county/district scales in China simultaneously. Second, lung cancer mortality continues to be higher in urban areas, though there appears to have been a reduction. Third, although lower than urban areas, lung cancer mortality appears to be rising in rural areas.

### 4.2. Comparisons with Other Studies

Lung cancer is the leading cancer typein males, comprising 17% of the total new cancer cases and 23% of the total cancer deaths [16,17]. The mortality rate among males is higher than females primarily due to the prevalence of cigarette smoking, which has historically been substantially higher among men than among women [18].

Our study revealed significant geographical variations in lung cancer mortality in China. The northeast region has the highest mortality rates and the northwest region has the lowest mortality rates. Previous research [19,20,21] has indicated that longer winter times with lower temperatures in the northeast area may lead to more indoor air pollution from unventilated coal-fueled stoves and cooking fumes. Primary factors closely tied to lung cancer in never-smokers include exposure to known and suspected carcinogens including radon, second-hand tobacco smoke, and other indoor air pollutants [22]. This might indicate why lung cancer mortality is highest in the northeast. In addition, a high prevalence of exposure to smoking [23] and particulate matter air pollution [24] will have adverse health effects relevant to lung cancer mortality. This study did not include relevant data, and we will explore it in further research.

Urbanization is a proxy for other risk factors for lung cancer mortality, such as air pollution. The higher lung cancer mortality in urban areas is consistent with previous findings [25]. With rapid economic development, the rate of urbanization has increased quickly in China. Urbanization is associated with development, but also has potential adverse health effects such as air pollution. Usually air pollution in rural areas is driven by combustion of solid fuels, while in the cities air pollution is derived from the influence of motor vehicles, both on primary emission and secondary formation [26]. It is plausible that the rapid urbanization of some rural areas in China may lead to an increased clustering of risk factors for lung cancer, which would help to explain the rising lung cancer mortality in rural areas observed in this study. Increases in rural areas may also be explained by poor access to preventive healthcare and treatment services. For example, from 2006, China initiated a policy to ban tobacco smoking, but this was mostly in urban areas [27].

### 4.3. Strengths and Limitations

This is the first study in which temporal and geographical variations in lung cancer mortality with representative data were investigated on a national scale in China. The data included 161 DSPs and all 31 mainland provinces. The data was adjusted for under-reporting [28]. In addition, the use of multilevel modeling was another important strength. The model afforded insights into temporal trends and geographic variations across multiple spatial scales. The study, however, is not without limitations. We lacked high-quality times-series data on air pollution, tobacco smoking, and related preventive healthcare and treatment services. It is likely that adjusting for those variables, had they been available, would have explained a considerable amount of the geographical variation observed (including the urbanization trends).

## 5. Conclusions

This study has demonstrated spatiotemporal variation in lung cancer mortality between 2006 and 2012. The findings of this study suggest that lung cancer mortality remained higher in the northeast than in other regions. Furthermore, although lung cancer mortality appears to have decreased in urban areas, the increase in rural areas is a cause for public health concern and warrants further investigation.

## Figures and Tables

**Table 1:** Incidence rate ratios and 95% confidence intervals for lung cancer mortality in China between 2006 and 2012 inclusive.

	Model 1	Model 2	Model 3
**Fixed effect**	Incidence Rate Ratio (95% Confidence Interval)		
Gender (ref: male)			
female	0.399 (0.393, 0.406) *	0.399 (0.393, 0.406) *	0.399 (0.393, 0.406) *
Age (5-year groups)	1.505 (1.502, 1.508) *	1.505 (1.502, 1.508) *	1.505 (1.499, 1.511) *
Time (years)	1.052 (1.034, 1.071) *	1.052 (1.034, 1.071) *	1.052 (1.034, 1.071) *
Time^2^ (years^2^)	0.992 (0.990, 0.994) *	0.992 (0.990, 0.994) *	0.992 (0.990, 0.994) *
% of urbanization (ref: low)			
Moderate		1.045 (0.911, 1.199)	1.046 (0.912, 1.200)
High		1.164 (1.021, 1.328) *	1.147 (1.006, 1.308) *
Region (ref: northwest)			
North			1.982 (1.212, 3.241) *
East			1.866 (1.187, 2.935) *
Central			1.866 (1.072, 3.250) *
South			1.361 (0.771, 2.402)
Southwest			1.026 (0.626, 1.682)
Northeast			2.442 (1.400, 4.262) *
**Random effects**			
Variance between provinces (standard error)	0.257 (0.071) *	0.248 (0.069) *	0.130 (0.039) *
MRR (provinces)	1.622	1.608	1.410
PCV (provinces)	-	3.50%	49.42%
Variance between counties/districts (standard error)	0.150 (0.020) *	0.135 (0.018) *	0.137 (0.019) *
MRR (counties/districts)	1.447	1.420	1.423
PCV (counties/districts)	-	10.00%	8.67%
Counties/districts slope variance by year (standard error)	0.003 (0.000) *	0.003 (0.000) *	0.003 (0.000) *
Covariance between DSP intercepts and slopes	−0.014 (0.003) *	−0.012 (0.002) *	−0.012 (0.002) *

* *p* < 0.05; MRR = median rate ratio; PCV = proportional change in variance in Model x compared to Model 1; DSP = disease surveillance points.
